# Cutaneous sensory nerve‐mediated microvascular vasodilation in normotensive and prehypertensive non‐Hispanic Blacks and Whites

**DOI:** 10.14814/phy2.14437

**Published:** 2020-05-13

**Authors:** Casey G. Turner, James T. Miller, Jeffrey S. Otis, Matthew J. Hayat, Arshed A. Quyyumi, Brett J. Wong

**Affiliations:** ^1^ Department of Kinesiology & Health Georgia State University Atlanta GA USA; ^2^ School of Public Health Georgia State University Atlanta GA USA; ^3^ Emory Clinical Cardiovascular Research Institute School of Medicine Emory University Atlanta GA USA

**Keywords:** human, nitric oxide, skin

## Abstract

Relative to non‐Hispanic Whites, non‐Hispanic Blacks are disproportionately affected by elevated blood pressure (BP). It is unknown whether race or subclinical increases in BP affect the ability of cutaneous sensory nerves to induce cutaneous microvascular vasodilation. Sixteen participants who self‐identified as non‐Hispanic Black (*n* = 8) or non‐Hispanic White (*n* = 8) were subgrouped as normotensive or prehypertensive. Participants were instrumented with three intradermal microdialysis fibers: (a) control, (b) 1 μM sodium nitroprusside (SNP), an exogenous nitric oxide (NO) donor, and (c) 20 mM N^G^‐nitro‐l‐arginine methyl ester (L‐NAME), a non‐selective NO synthase inhibitor. A slow local heating protocol (33–40°C, 0.1°C/min) was used to assess the onset of cutaneous sensory nerve‐mediated vasodilation (temperature threshold) and skin blood flow was measured using laser‐Doppler flowmetry. At control sites, the temperature threshold occurred at a higher temperature in non‐Hispanic Blacks (normotensive: 37.2 ± 0.6°C, prehypertensive: 38.9 ± 0.5°C) compared to non‐Hispanic Whites (normotensive: 35.2 ± 0.8°C, prehypertensive: 35.2 ± 0.9°C). L‐NAME shifted the temperature threshold higher in non‐Hispanic Whites (normotensive: 37.8 ± 0.7°C, prehypertensive: 38.2 ± 0.8°C), but there was no observed effect in non‐Hispanic Blacks. SNP did not affect temperature threshold in non‐Hispanic Whites, but shifted the temperature threshold lower in non‐Hispanic Blacks (normotensive: 34.6 ± 1.2°C, prehypertensive: 34.8 ± 1.1°C). SNP mitigated differences in temperature threshold across all groups. There was no effect found for BP status in either the non‐Hispanic Black or non‐Hispanic White groups. These data suggest that reduced NO bioavailability affects the ability of cutaneous sensory nerves to induce microvascular vasodilation in young, otherwise healthy non‐Hispanic Blacks.

## Graphical abstract

Cutaneous sensory nerve activation is shifted to a higher temperature threshold in non‐Hispanic Blacks relative to non‐Hispanic Whites. Administration of exogenous nitric oxide via nitroprusside lowered the temperature threshold in non‐Hispanic Blacks but not in non‐Hispanic Whites.

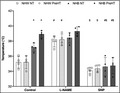

## 1 INTRODUCTION

Hypertension, an established cardiovascular disease (CVD) risk factor, affects roughly 46% of the US adult population (Benjamin et al., 2019). The prevalence of hypertension is greater in non‐Hispanic Blacks relative to non‐Hispanic Whites (Benjamin et al., 2019). Prevalence rates of prehypertension are often underreported clinically, but are estimated to be similar to rates of hypertension, further suggesting an increased prevalence of prehypertension in non‐Hispanic Blacks. Prehypertension is the subclinical elevation of blood pressure (BP) (systolic between 120 and 129 mmHg or diastolic between 80 and 89 mmHg; [Whelton et al., 2017]) and is a critical pivot point in long‐term health. Prehypertension is linked to subclinical manifestions of coronary atherosclerosis (Erdogan et al., 2007) and is an independent risk factor for hypertension and CVD (Weil, Westby, Greiner, Stauffer, & DeSouza, 2012). Furthermore, prehypertensive non‐Hispanic Blacks are 35% more likely to develop overt hypertension than prehypertensive non‐Hispanic Whites (Selassie et al., 2011). Non‐Hispanic Blacks may be at greater risk of developing elevated BP due to altered vasodilator mechanisms.

Cutaneous microvascular function is used as a general model of systemic microvascular function (Holowatz, Thompson‐Torgerson, & Kenney, 2008). The cutaneous microvasculature is easily accessible and can be studied with minimally invasive techniques (Johnson & Kellogg, 2010; Johnson, Minson, & Kellogg, 2014), such as locally heating the skin and measuring the skin blood flow response (Houghton, Meendering, Wong, & Minson, 2006; Minson, Berry, & Joyner, 2001). The cutaneous sensory nerves initate the cutaneous microvascular vasodilator response to local, non‐painful heating (Minson et al., 2001). The specific temperature at which cutaneous sensory nerve activation occurs can be assessed by slow local heating (0.1°C/min) of the skin (Choi, Brunt, Fujii, & Minson, 2014; Hodges, Kosiba, Zhao, & Johnson, 2008; Hodges, McGarr, Mallette, Pozzi, & Cheung, 2016; Houghton et al., 2006). It is currently unknown whether the ability of cutaneous sensory nerves to induce cutaneous microvascular vasodilation is disrupted in at‐risk populations, such as non‐Hispanic Blacks or prehypertensive individuals.

The detrimental effect of other clinical diseases (e.g., type II diabetes mellitus and cancer) on sensory nerve activation is well‐known, but the relationship between elevated BP and sensory nerve activation is largely unknown. Data suggest that overt hypertension is an independent risk factor for sensory neuropathy (Cohen, Jeffers, Faldut, Marcoux, & Schrier, 1998; Emdin, Anderson, Woodward, & Rahimi, 2015; Hebert, Veluchamy, Torrence, & Smith, 2017; Tesfaye et al., 2005), though fundamental mechanisms are not fully understood at this time. The effect of subclinical prehypertension on sensory nerve activation is unknown. Sensory neuropathy involves damage or dysfunction of the sensory nerves, and the pathophysiology is linked to microvascular and endothelial dysfunction (Cameron, Eaton, Cotter, & Tesfaye, 2001). Microvascular and endothelial dysfunction also accompany prehypertension (Erdogan et al., 2007; Murgan et al., 2013; Weil, Stauffer, Greiner, & DeSouza, 2011; Weil et al., 2012). Subsequently, sensory nerve dysfunction may further exacerbate vascular dysfunction, as sensory nerve neuropeptide release contributes to BP modulation via alterations in peripheral tone (Watson, Supowit, Zhao, Katki, & DiPette, 2002). The relationship between race, BP status, and sensory nerve function is currently unclear.

Basal levels of nitric oxide (NO) are required for full activation of the cutaneous sensory nerves and sub‐basal levels may lead to reduced sensory nerve activation or reduced sensitivity to stimuli (Houghton et al., 2006; Minson et al., 2001), such as thermal stimuli. Relative to non‐Hispanic Whites, non‐Hispanic Blacks show reduced NO‐dependent vasodilation (Hurr, Patik, Kim, Christmas, & Brothers, 2018; Kim, Hurr, Patik, & Brothers, 2018; Ozkor et al., 2014; Patik, Curtis, Akins, et al., 2018; Patik, Curtis, Nasirian, et al., 2018), reduced antioxidant capacity (Morris et al., 2012), and increased oxidative stress (Deo, Holwerda, Keller, & Fadel, 2015; Feairheller et al., 2011; Hurr et al., 2018; Morris et al., 2012). These factors suggest reduced NO bioavailability in non‐Hispanic Blacks (Brothers, Fadel, & Keller, 2019; Jin & Loscalzo, 2010). Similar differences are noted between normotensive and hypertensive inidivuals (Holowatz & Kenney, 2007a, 2007b; Smith et al., 2011), but it is unknown if differences in NO bioavailability exist between normotensive and prehypertensive individuals. Reduced NO bioavailbility may therefore be a mechanism through which sensory nerve activaition is affected in non‐Hispanic Blacks and/or prehypertensive individuals.

The purpose of this study was to determine whether race and BP status affect the onset of cutaneous sensory nerve‐mediated vasodilation. We hypothesized that (a) cutaneous sensory nerve activation would occur at a higher temperature threshold in non‐Hispanic Blacks compared to non‐Hispanic Whites, suggesting delayed cutaneous sensory nerve activation, (bb) prehypertension would further delay cutaneous sensory nerve activation in non‐Hispanic Blacks but not non‐Hispanic Whites, and (c) a low dose of exogenous NO would abolish differences in cutaneous sensory nerve activation between groups.

## 2 METHODS

### 2.1 Ethical approval

All participants provided written and verbal informed consent. Advarra Institutional Review Board (Columbia, MD) and the Georgia State University Institutional Review Board approved this study (Pro00024265). The use of all pharmacological agents was approved by the Food and Drug Administration (IND 138231). This study conformed to the guidelines set forth by the *Declaration of Helsinki.*

### 2.2 Participants

Participant demographics are shown in Table 1. All participants self‐identified as non‐Hispanic Black or non‐Hispanic White. Participants were then further divided into groups based on BP status: normotensive (systolic <120 mmHg and diastolic <80 mmHg) or prehypertensive (systolic between 120 and 129 mmHg or diastolic between 80 and 89 mmHg), based on the 2017 American Heart Association guidelines (Whelton et al., 2017). Participants completed a health history questionnaire and were not taking medications (other than oral contraceptive pills; non‐Hispanic White = 3, non‐Hispanic Black = 2) or supplements, were nonsmokers, and had no history of known cardiovascular, metabolic, and neurologic diseases. Participants were asked to avoid alcohol, excessive caffeine, strenuous exercise, and high fat meals for at least 8 hr prior to the experiment. Female participants not on oral contraceptives took a urine pregnancy test (McKesson hCG Combo Test Casssette, Consult Diagnostics) to confirm negative pregnancy status. Menstrual cycle phase was recorded, but not controlled for, in these studies. Although the effect of oral contraceptives (exogenous sex hormones) are known to influence the cutaneous vascular response to *rapid* local heating (Charkoudian, Stephens, Pirkle, Kosiba, & Johnson, 1999), it is not known whether exogenous female sex hormones influence the response to *slow* local heating as employed in the present study. To date, the influence of endogenous female sex hormones on cutaneous microvascular function is not clearly delineated (Cracowski, 2011). In addition, it is not well‐established whether testing female participants when estrogen levels are low is always appropriate. Therefore, to increase generalizability, we included males and females, as well as females at any phase of the menstrual cycle/oral contraceptive use in the present study.

**Table 1:** Summary statistics for study participants

	Non‐Hispanic Black		Non‐Hispanic White	
	Normotensive (*n* = 4)	Prehypertensive (*n* = 4)	Normotensive (*n* = 4)	Prehypertensive (*n* = 4)
Age (years)	24 ± 4 (18–28)	22 ± 2 (19–24)	27 ± 7 (21–36)	26 ± 6 (20–33)
Male/female (#)	1/3	2/2	2/2	2/2
Menstrual cycle/OC phase				
Menstrual/placebo	2 (1 OC)	1 (1 OC)	1 (1 OC)	
Luteal/high hormone	1	1	1 (1 OC)	2 (1 OC)
Height (cm)	173.0 ± 3.7	167.5 ± 6.0	169.8 ± 12.9	173.8 ± 11.5
Mass (kg)	77.0 ± 16.2	83.2 ± 7.9	77.5 ± 10.7	72.1 ± 10.1
BMI (kg/m^2^)	22.2 ± 4.4	24.9 ± 2.9	22.8 ± 2.0	20.7 ± 2.4
HR (bpm)	75 ± 18	67 ± 13	64 ± 7	68 ± 7
SBP (mmHg)	110 ± 6 (104–116)	124 ± 5 (120–129)	109 ± 9 (100–118)	122 ± 1 (121–123)
DBP (mmHg)	68 ± 5 (64–72)	74 ± 3 (70–75)	69 ± 5 (64–76)	78 ± 2 (77–80)
MAP (mmHg)	82 ± 4 (76–86)	91 ± 2 (88–93)	83 ± 6 (76–91)	92 ± 7 (91–94)

Note

Data shown are mean ± *SD*. Blood pressure data includes the range in parentheses.

Abbreviations: BMI, body mass index; DBP, diastolic blood pressure; HR, heart rate; MAP, mean arterial pressure; OC, oral contraceptive; SBP, systolic blood pressure.

### 2.3 Instrumentation

All trials were conducted in a temperature‐controlled (22°C, 38% rh) laboratory at Georgia State University. Mass in kilograms and height in centimeters were obtained using a digital scale and platform stadiometer (Healthometer Professional, Pelstar). Participants sat in a semirecumbent chair with experimental arm at approximate heart level for the entirety of the protocol. Three microdialysis fibers (CMA 31 Linear Microdialysis Probe, 55 kDa cut‐off membrane; CMA microdialysis AB) were placed on the *dorsal aspect of the left forearm* (Fujii et al., 2019; Mack, Foote, & Nelson, 2016) with at least 4 cm separating each fiber. An ice pack was used to numb the skin prior to fiber placement (Hodges, Chiu, Kosiba, Zhao, & Johnson, 2009). A 23‐gauge needle was initially inserted into the dermal layer of the skin. Microdialysis fibers were threaded through the lumen of the needle and the semi‐permeable membrane was drawn into place and left under the skin during needle removal. Fibers were then taped to the skin. All experimental drugs were perfused using a microinfusion pump (BASi Bee Hive) at a rate of 2 µl/min.

Blood pressure measurements were obtained on the contralateral arm via automated brachial auscultation every 10 min (Welch Allyn Vital Signs Series 6000). Mean arterial pressure (MAP) was calculated as one‐third pulse pressure plus diastolic pressure. Red blood cell (RBC) flux, an index of skin blood flow, was measured over each microdialysis fiber site using laser‐Doppler flowmetry (VP7 A/T with Moor VMS‐LDF2; Moor Instruments). Each laser‐Doppler probe was held within the center of a local heating element to control skin temperature throughout the protocol (Moor‐HEAT; Moor Instruments).

### 2.4 Experimental protocol

Following microdialysis fiber placement, trauma from needle insertion was allowed to resolve for approximately 30–60 min, but was extended as necessary for erythema or minor swelling to resolve. Trauma resolution was facilitated by microinfusion of lactated Ringer's solution (Baxter Healthcare) through all three sites. Sites were randomly assigned an experimental treatment and were then perfused with the corresponding experimental agent for 45–60 min before local heating: (a) lactated Ringer's to serve as a control site, (b) 1 µM sodium nitroprusside (SNP; EMD Millipore Corp; [Houghton et al., 2006]), an exogenous NO donor, and (c) 20 mM N^G^‐nitro‐l‐arginine methyl ester (L‐NAME; EMD Millipore Corp; [Yamazaki et al., 2006]), a nonselective NO synthase inhibitor. Local heaters were set to 33°C to maintain normal skin temperature during baseline (~15 min). Local heaters were then raised to 40°C using a slow local heating protocol, increasing at a rate of 0.1°C/min (Choi et al., 2014; Hodges et al., 2008, 2016; Houghton et al., 2006). A stable plateau was obtained at 40°C for approximately 10 min. Finally, local heaters were increased to 43°C and 54 mM SNP was infused to induce maximal cutaneous vasodilation (Holowatz, Thompson, Minson, & Kenney, 2005; McNamara, Keen, Simmons, Alexander, & Wong, 2014; Minson et al., 2001; Wong & Fieger, 2010).

### 2.5 Data analysis

Continuous RBC flux data were collected at 100 Hz using Power Lab data acquisition hardware (ADI Instruments) and Lab Chart software (ADI Instruments). Cutaneous vascular conductance (CVC) was calculated as RBC flux divided by MAP. Maximal blood flow data are presented as CVC. Baseline data are presented as a percentage of maximal CVC (%CVC_max_) obtained during maximal cutaneous vasodilation at the end of the protocol.

The slow local heating protocol was allowed for assessment of temperature threshold for the onset of sensory nerve‐mediated vasodilation. This was quantified as the temperature to the nearest 0.1°C which produced an axon reflex within the heating protocol (33.0–40.0°C; [Houghton et al., 2006]). An axon reflex was categorized as an increase in RBC flux of at least 10 units (Houghton et al., 2006) that was not otherwise attributable to movement of the participant or other disturbances. To minimize bias when identifying an axon reflex response, data from each participant were exported from Lab Chart to Microsoft Excel without any identification of participant race or BP status and without any indication of microdialysis site treatment. Axon reflex responses were independently analyzed by two of the authors. Of the 48 observations, the two investigators agreed on 91.6% (44/48) axon reflex responses (difference of <0.1°C between the two investigators); for the remaining four observations the difference between investigators was 0.2°C and, in these cases, the assessment of the more senior investigator was used for analysis.

### 2.6 Statistical analysis

Temperature threshold and CVC data were compared using a three‐way ANOVA with factors of race (non‐Hispanic Black and non‐Hispanic White), BP status (normotensive and prehypertensive), and microdialysis treatment (control, L‐NAME, and SNP). Tukey's post hoc test was used to control for multiple comparisons. All statistical analyses and graphs were completed using commercial software (SAS; GraphPad Prism 8). A priori power analyses were conducted. The sample sizes for each group were calculated based on effect sizes observed in previous studies in our lab. Assuming a 0.05 level of significance, 80% power, and a temperature threshold of difference between groups of 1.3°C (*SD* = 0.2–0.6), the needed sample size was 4 in each group. All data are presented as mean ± *SD* and a level of significance of 0.05 was used for statistical significance. To emphasize physiological significance over statistical significance, *p *> .05 but <0.10 were deemed to represent *physiologically significant differences* (Curran‐Everett, 2009; Drummond, 2020; Hayat, 2010).

## 3 RESULTS

Participant demographics are presented in Table 1. By design, systolic BP was lower in normotensive groups than prehypertensive groups.

Baseline %CVC_max_ and absolute maximal CVC values are presented in Table 2. Administation of low dose SNP significantly increased baseline skin blood flow compared to respective control and L‐NAME sites in normotensive non‐Hispanic Blacks and respective L‐NAME sites in prehypertensive non‐Hispanic Whites. There were no observed differences in maximal absolute CVC values between groups or treatment sites.

**Table 2:** Summary statistics for baseline and maximal skin blood flow responses across microdialysis treatment groups

	Non‐Hispanic Black		Non‐Hispanic White	
	Normotensive (*n* = 4)	Prehypertensive (*n* = 4)	Normotensive (*n* = 4)	Prehypertensive (*n* = 4)
Baseline (%CVC_max_)				
Control	14 ± 8	13 ± 5	15 ± 5	19 ± 9
L‐NAME	10 ± 2	17 ± 11	11 ± 4	11 ± 4
SNP	28 ± 4*	13 ± 6	19 ± 8	35 ± 14^#^
Maximal (CVC)				
Control	2.82 ± 0.71	2.59 ± 0.48	3.12 ± 0.65	2.96 ± 0.31
L‐NAME	2.48 ± 0.93	1.87 ± 0.20	2.37 ± 0.25	2.27 ± 0.41
SNP	2.88 ± 0.55	2.74 ± 0.57	2.79 ± 0.44	2.60 ± 0.43

Abbreviations: CVC, cutaneous vascular conductance; L‐NAME, N^G^‐nitro‐l‐arginine methyl ester; SNP, sodium nitroprusside.

* *p* < .05 versus control

^#^ *p* < .05 versus L‐NAME.

**FIGURE 1:**
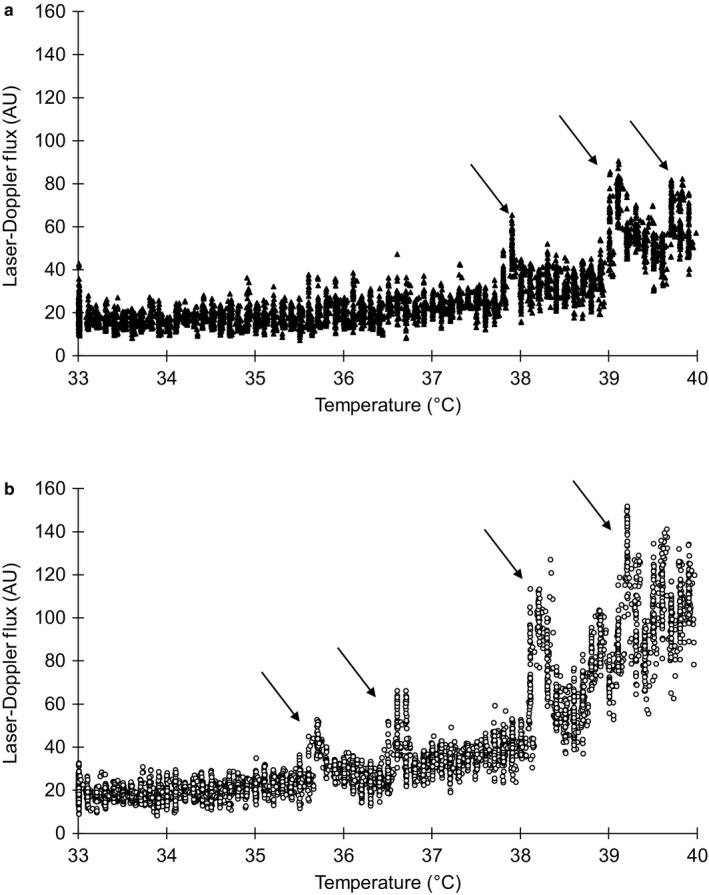
(a and b) Representative tracing of the skin blood flow response to gradual local heating at the control site in one non‐Hispanic Black normotensive participant (a, top tracing) and one non‐Hispanic White normotensive participant (b, bottom tracing). Downward arrows indicate the first observed axon reflex response. *Y*‐axis values are laser‐Doppler flux units in order to magnify the axon reflex responses. For clarity, tracings for prehypertensive participants and for L‐NAME and SNP sites are not shown. L‐NAME, N^G^‐nitro‐l‐arginine methyl ester; SNP, sodium nitroprusside.

A representative tracing from the control site in one non‐Hispanic Black‐normotensive participant and one non‐Hispanic White‐normotensive participant is shown in Figure 1. The tracing depicts the onset of axon reflex responses (arrows) occurring at a lower heater temperature in the non‐Hispanic White participant compared to the non‐Hispanic Black participant. For clarity, responses for prehypertensive participants and those for L‐NAME and SNP sites are not shown.

### 3.1 Temperature threshold data

**FIGURE 2:**
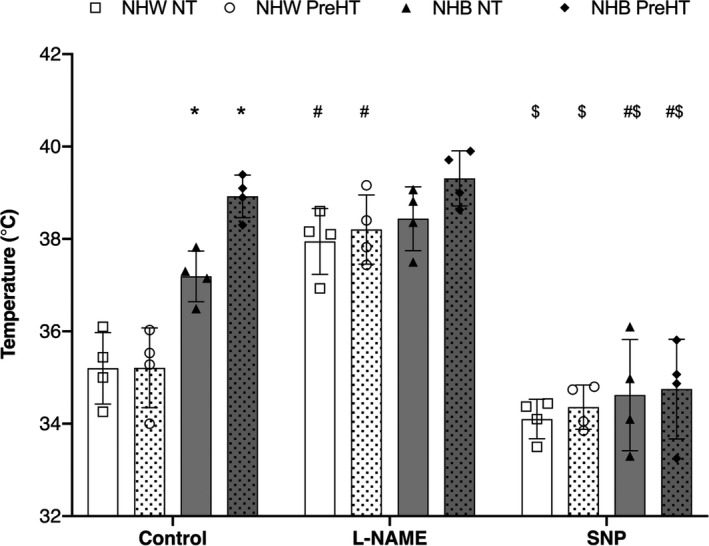
Temperature threshold data (°C) as mean ± *SD*. L‐NAME, N^G^‐nitro‐l‐arginine methyl ester; SNP, sodium nitroprusside. *N* = 4 per group. Individual data points are included for each experimental site: non‐Hispanic White normotensive (open squares, open bars), non‐Hispanic White prehypertensive (open circles, white dotted bars), non‐Hispanic Black normotensive (closed triangles, solid gray bars), non‐Hispanic Black prehypertensive (closed diamonds, gray dotted bars). *p < .05 versus non‐Hispanic White normotensive and non‐Hispanic White prehypertensive at control sites, ^#^ *p* < .05 versus respective control sites, ^$^ *p* < .05 versus respective L‐NAME sites

Temperature threshold data are presented in Figure 2. An increase in temperature threshold is an indication of later onset of cutaneous sensory nerve‐mediated vasodilation.

At control sites, temperature threshold was significantly higher in non‐Hispanic Black (normotensive: 37.2 ± 0.6°C, prehypertensive: 38.9 ± 0.5°C) compared to non‐Hispanic White (normotensive: 35.2 ± 0.8°C, prehypertensive: 35.2 ± 0.9°C) regardless of BP status. There was no difference found between normotensive and prehypertensive non‐Hispanic Whites at control sites. Although not statistically significant, there was a physiologically significant difference between normotensive and prehypertensive non‐Hispanic Blacks (ΔT = 1.7°C; *p* = .0859).

Compared to control sites, treatment with L‐NAME significantly increased temperature threshold in non‐Hispanic White groups (normotensive: 37.8 ± 0.7°C, *p* = .0006; prehypertensive: 38.2 ± 0.8°C, *p* = .0001), but did not alter the temperature threshold in non‐Hispanic Black groups (normotensive: 38.4 ± 0.7°C, *p* = .4648; prehypertensive: 39.3 ± 0.6°C, *p* = .9998).

Treatment with SNP did not alter temperature threshold in non‐Hispanic White groups (normotensive: 34.1 ± 0.4°C, *p* = .6492; prehypertensive: 34.4 ± 0.5°C, *p* = .8984), but significantly decreased temperature threshold in non‐Hispanic Black groups (normotensive: 34.6 ± 1.2°C, *p* = .0013; prehypertensive: 34.8 ± 1.1°C, *p* < .0001) compared to control sites. Temperature thresholds at SNP sites were also reduced compared to L‐NAME sites in all groups (*p* < .0001 for all comparisons).

There were no differences between groups at either the L‐NAME or SNP sites. Exact *p* values for all between group comparisons are shown in Table 3.

**Table 3:** *p* values for between group comparisons

	*p* value
Control site comparisons	
Normotensive non‐Hispanic White versus	
Prehypertensive non‐Hispanic White	>.9999
Normotensive non‐Hispanic Black	.0267
Prehypertensive non‐Hispanic Black	<.0001
Prehypertensive non‐Hispanic White	
Normotensive non‐Hispanic Black	.0280
Prehypertensive non‐Hispanic Black	<.0001
Normotensive non‐Hispanic Black versus Prehypertensive non‐Hispanic Black	.0859
L‐NAME site comparisons	
Normotensive non‐Hispanic White versus	
Prehypertensive non‐Hispanic White	>.9999
Normotensive non‐Hispanic Black	.9984
Prehypertensive non‐Hispanic Black	.3365
Prehypertensive non‐Hispanic White	
Normotensive non‐Hispanic Black	>.9999
Prehypertensive non‐Hispanic Black	.6400
Normotensive non‐Hispanic Black versus Prehypertensive non‐Hispanic Black	.8819
SNP site comparisons	
Normotensive non‐Hispanic White versus	
Prehypertensive non‐Hispanic White	>.9999
Normotensive non‐Hispanic Black	.9974
Prehypertensive non‐Hispanic Black	.9839
Prehypertensive non‐Hispanic White	
Normotensive non‐Hispanic Black	>.9999
Prehypertensive non‐Hispanic Black	.9998
Normotensive non‐Hispanic Black versus prehypertensive non‐Hispanic Black	>.9999

## 4 DISCUSSION

The main findings of this study were: (a) cutaneous sensory nerve activation occurs at a higher temperature in non‐Hispanic Blacks compared to non‐Hispanic Whites, (b) in contrast with our second hypothesis, we did not observe an effect of BP status on temperature threshold, and (c) administration of exogenous NO via low‐dose SNP abolished differences in cutaneous sensory nerve activation between all groups.

The majority of local heating studies use a rapid local heating protocol (0.1°C/s) to assess cutaneous microvascular vasodilation (Johnson & Kellogg, 2010; Minson et al., 2001). The cutaneous microvascular vasodilator response to rapid local heating of the skin is biphasic with cutaneous sensory nerves largely mediating the initial onset of vasodilation (i.e., the initial peak; [Hodges, Pozzi, McGarr, Mallette, & Cheung, 2015; Minson et al., 2001]). Blockade of the sensory nerves via topical anesthetic (EMLA) treatment greatly reduces, but does not abolish, the initial vasodilation in response to rapid local heating (Hodges et al., 2015) and also delays the onset in response to slow local heating (0.1°C/min; [Hodges et al., 2016]). A limited number of studies have used a slow local heating protocol to more specifically assess the temperature at which sensory nerve‐mediated vasodilation begins (Choi et al., 2014; Hodges et al., 2008, 2016; Houghton et al., 2006), represented by significant, but brief, increases in skin blood flow (depicted in Figure 1a,b; [Houghton et al., 2006]). The present study was aimed to assess the effect of race and BP status, in relation to NO bioavailability, on the temperature of sensory nerve‐mediated vasodilation onset.

A higher temperature threshold for the onset of cutaneous sensory nerve‐mediated vasodilation was observed at control sites of non‐Hispanic Black compared to non‐Hispanic White groups, suggestive of delayed cutaneous sensory nerve activation in non‐Hispanic Blacks. NO synthase inhibition with L‐NAME delayed the onset of cutaneous vasodilation in non‐Hispanic Whites but not non‐Hispanic Blacks, indicating the importance of NO to sensory nerve activation and the presence of reduced NO bioavailability in non‐Hispanic Blacks. Conversely, the addition of exogenous NO with low‐dose SNP shifted the onset of cutaneous vasodilation to a lower temperature in non‐Hispanic Blacks but not non‐Hispanic Whites, again indicating NO decrements in non‐Hispanic Black but not non‐Hispanic White individuals. The difference between groups was abolished following the administration of an equal dose of SNP across groups. Because of this, the delay in cutaneous sensory nerve activation and onset of cutaneous sensory nerve‐mediated vasodilation in non‐Hispanic Blacks appears to be attributable to reduced NO bioavailability and not reduced sensory nerve sensitivity to NO.

A basal level of NO is required for full activation of sensory nerves (Houghton et al., 2006; Kellogg, Liu, Kosiba, & O’Donnell, 1999; Minson et al., 2001; Minson, Holowatz, Wong, Kenney, & Wilkins, 2002). Furthermore, it appears a basal level of *endogenous* NO present prior to heating may be of primary importance, as combined infusion of L‐NAME and low‐dose SNP (*exogenous* NO donor) abolished sensory nerve‐mediated vasodilation similarly to L‐NAME infusion alone (Houghton et al., 2006). NO may contribute to the onset of sensory nerve‐mediated vasodilation through several pathways. First, NO mediates the action of two potent vasodilators, calcitonin gene‐related peptide (CGRP; [Hoon, Pickkers, Smits, Struijker‐Boudier, & Van Bortal, 2003]) and substance P (Morioka et al., 2002). CGRP and substance P are colocalized in sensory nerve terminals and both elicit antidromic vasodilation (Sann & Pierau, 1998; Wallengren & Hakanson, 1987), but the exact role of either neuropeptide in sensory nerve‐mediated vasodilation is not completely understood. Second, transient receptor vanilloid potential (TRPV) channels, primarily TRPV‐1 and TRPV‐4, are located on afferent sensory nerves (Caterina, 2007; Lin, Li, Xu, Zou, & Fang, 2007; Vriens, Appendino, & Nilius, 2009; Wu et al., 2007) and have been implicated in the initiation of cutaneous vasodilation (Mack et al., 2016; Wong & Fieger, 2010) and may increase the production of NO (Yao & Garland, 2005). Third, data also suggests that NO mediates the potentiation of TRPV channels (Miyamoto, Dubin, Petrus, & Patapoutian, 2009; Yao & Garland, 2005; Yoshida et al., 2006). Collectively, reduced NO bioavailability may affect the onset of sensory nerve‐mediated vasodilation by affecting one or more of these potential pathways.

Reduced NO bioavailability (Jin & Loscalzo, 2010; Kim et al., 2018; Ozkor et al., 2014) and factors that influence NO bioavailability, such as high oxidant stress (Hurr et al., 2018; Patik, Curtis, Nasirian, et al., 2018), increased ROS production (Deo et al., 2015; Feairheller et al., 2011; Kalinowski, Dobrucki, & Malinski, 2004), and reduced antioxidant mechanisms (Morris et al., 2012), have all been indicated in young, otherwise healthy non‐Hispanic Blacks. Superoxide appears specifically impactful on NO bioavailability in non‐Hispanic Blacks (Hurr et al., 2018; Patik, Curtis, Nasirian, et al., 2018). To date, one study has reported data on the initial vasodilatory peak in response to rapid local heating between non‐Hispanic Black and non‐Hispanic White groups (Patik, Curtis, Nasirian, et al., 2018). The initial peak was reduced in non‐Hispanic Black individuals compared to non‐Hispanic White individuals and was increased, specifically in non‐Hispanic Black males only, through the inhibition of superoxide producing enzymes, xanthine oxidase and NADPH oxidase (Patik, Curtis, Nasirian, et al., 2018). Increased superoxide production or reduced superoxide dismutase activity (Feairheller et al., 2011) may be critical mechanisms that affect the onset of cutaneous sensory nerve‐mediated vasodilation in non‐Hispanic Blacks.

Temperature threshold values recorded in this study differ slightly from other slow local heating studies. A limited number of slow local heating studies have been completed and those studies report control site temperature thresholds between 35.3°C and 38.1°C (Choi et al., 2014; Hodges et al., 2008, 2016; Houghton et al., 2006). Magerl & Treede (1996) suggested warm‐sensitive nociceptors induce cutaneous sensory nerve‐mediated vasodilation, implying cutaneous sensory nerves do not elicit microvascular vasodilation to non‐painful heating at temperatures <35°C (Magerl & Treede, 1996). However, the present data demonstrates sensory nerve activation at temperatures <35°C in SNP sites (34.4–34.8°C). These data suggest that warm afferents may contribute to the initiation of cutaneous sensory nerve‐mediated vasodilation during slow local heating or following full activation with the addition of exogenous NO. TRPV channels have been implicated in the induction of cutaneous vasodilation (Fujii et al., 2019; Mack et al., 2016; Wong & Fieger, 2010). TRPV‐3 and TRPV‐4 channels are expressed in human keratinocytes and are presumed to detect warm temperature stimuli below temperatures of 35°C (Fujii et al., 2019; Schepers & Ringkamp, 2009). TRPV‐4 and TRPV‐3 channels have been shown to be activated by NO (Fujii et al., 2019; Yoshida et al., 2006) and to increase NO production (Miyamoto, Petrus, Dubin, & Patapoutian, 2011; Seth et al., 2017). Therefore, warm afferents via TRPV channels may contribute to initial mechanisms of cutaneous sensory nerve‐mediated vasodilation and, as such, may be paramount in understanding discrepancies between racial groups.

### 4.1 Clinical perspectives

Microvascular dysfunction is apparent in hypertensive individuals (Holowatz & Kenney, 2007a, 2007b). Few studies have investigated the effect of prehypertension on microvascular vasodilation, but the presence of microvascular and endothelial dysfunction in other microvascular beds is supported (Erdogan et al., 2007; Murgan et al., 2013; Weil et al., 2011, 2012). Individuals with prehypertension are more likely to develop overt hypertension (Leitschuh, Cupples, Kannel, Gagnon, & Chobanian, 1991; Winegarden, 2005), especially prehypertensive non‐Hispanic Blacks (Selassie et al., 2011). These studies suggest parallel pathophysiology from prehypertension to hypertension; therefore, decrements in cutaneous microvascular vasodilation may be measurable prior to the development of overt hypertension, as microvascular dysfunction often precedes macrovascular dysfunction (Cohuet & Struijker‐Boudier, 2006; Gutierrez et al., 2013; Mohammedi et al., 2017; Vallance & Chan, 2001; Verma, Buchanan, & Anderson, 2003).

Hypertension is also an apparent risk factor for sensory neuropathy (Cohen et al., 1998; Emdin et al., 2015; Hebert et al., 2017; Tesfaye et al., 2005). Data indicate pharmacological reduction in BP results in increased nerve conduction velocities (Hotta et al., 1999; Malik et al., 1998; Reja, Tesfaye, Harris, & Ward, 1995). Reduced NO bioavailability (Holowatz & Kenney, 2007a; Taddei, Virdis, Ghiadoni, Magagna, & Salvetti, 1997) and elevated oxidative stress (Holowatz & Kenney, 2007a; Lassegue & Griendling, 2004; Smith et al., 2011) are hallmarks of hypertension and greatly effect microvascular and endothelial function (Holowatz & Kenney, 2007a, 2007b). Existing microvascular dysfunction and reduced NO bioavailability can effectively reduce blood flow and oxygen delivery to sensory nerve endings, resulting in slowed neurogenic conductance (Cameron et al., 2001). The results of this study suggest effects of reduced NO bioavailability that reach beyond the direct vasculature to other structures (i.e., the sensory nerves), revealing other vulnerable aspects of the vasodilator response.

We did not observe an effect (statistical or physiological) of prehypertension in non‐Hispanic Whites, but we did observe a physiological (but not statistical) effect in prehypertensive non‐Hispanic Blacks. These findings suggest that subclinical increases in BP begin to exert detrimental effects on cutaneous sensory nerve activation in non‐Hispanic Blacks. It is possible that overt hypertension, rather than prehypertension, exerts detrimental effects on cutaneous sensory nerve function in non‐Hispanic Whites. The detrimental effect of reduced NO bioavailability on the cutaneous sensory nerves may also contribute to the increased conversion rate (Selassie et al., 2011) of prehypertension to hypertension in non‐Hispanic Blacks.

### 4.2 Limitations

The a priori sample size calculation resulting in *n* = 4 per group remains a small sample size for human‐based research studies. While appropriate statistical techniques were used to calculate sample size, this should be taken into consideration. The present study was completed in young, healthy individuals and, thus, cannot be generalized to age groups outside of 18–40 years old or to acutely or chronically diseased individuals. To increase generalizability to general young, healthy populations, we included males and females, as well as females at any phase of the menstrual cycle/oral contraceptive use in this present study. To date, the influence of endogenous female sex hormones on cutaneous microvascular function are not clearly delineated (Cracowski, 2011). It is possible that menstrual cycle phase impacts cutaneous microvascular function. Finally, though all participants completed a comprehensive self‐report health history questionnaire, objective screening blood analyses to ensure absence of hypercholesterolemia, hyperglycemia, etc. were not completed.

## 5 CONCLUSION

In conclusion, this is the first study to demonstrate that cutaneous sensory nerves are activated at a higher local temperature in young, healthy non‐Hispanic Black participants relative to young, healthy non‐Hispanic White participants. In non‐Hispanic Black, administration of low‐dose exogenous NO via SNP shifted the threshold for sensory nerve activation to a temperature similar to that observed for non‐Hispanic White, suggesting reduced NO bioavailability, and not reduced NO sensitivity, mediates the higher activation temperature at control sites. The findings of altered cutaneous sensory nerve‐mediated vasodilator mechanisms may contribute, in part, to the observed increased rates of prehypertension and hypertension in the non‐Hispanic Black population.

## CONFLICT OF INTEREST

The authors have no conflicts of interest to report.

## AUTHOR CONTRIBUTIONS

Casey Turner was responsible for data collection, data analysis and interpretation, and drafting all versions of the manuscript. James Miller was responsible for data collection, data interpretation, and editing all versions of the manuscript. Jeffrey Otis was responsible for experimental design, data interpretation, and editing all drafts of the manuscript. Matthew Hayat was responsible for conducting all statistical analyses and editing all drafts of the manuscript. Arshed Quyyumi was responsible for experimental design, data interpretation, and editing all drafts of the manuscript. Brett Wong was responsible for experimental design, data analysis and interpretation, and editing all drafts of the manuscript.
